# Isn’t It Pathetic?

**Published:** 1911-03-15

**Authors:** James W. Cormany

**Affiliations:** Mt. Caroll, Ill.


### ISN'T IT PATHETIC?

BY DR. JAMES W. CORMANY, MT. CAROLL, ILL.

I would like to plunge right into my subject, but I must make a little explanation as to what led me up to speak upon such a subject and why I do not take the more practical side of dentistry and talk about that.

I did write a paper once for this Society upon the subject of "Prosthetic Dentistry" and I have been sorry ever since. Its title was, Get the Case if You Can, Make a Fit if You Can, Get Your Pay if You Can." How is that for a title?

I think it necessary that other subjects than operative and prosthetic dentistry should be brought before this body. We need encouragement along other lines, a mental stimulus, if you please, and while the title of this paper sounds a little pessimistic I'll show you before I get through that I am as far away from that as the east is from the west.

I trust my paper is timely for we hear quite a little these days of the dissatisfaction existing among the fraternity caused no doubt, by the great desire for wealth, more wealth, more coin, more of everything good, bad or indifferent. And this leads me up to my subject.

What I am about to read was taken from the *Dental Brief*, and reads as follows; I presume addressed to the editor:

"Isn't It Pathetic?"

"My Dear Sir:—Do you think I could get you to help me out personally? I have done some advertising in the past in the newspapers, but it does not seem to help.

"There are eight dentists here in a town of 7,500, and three of us are old dentists. I have been located here for thirty years and have met with success until the last year or two. I can see that my business is dropping off in spite of my best efforts to keep it up.

"I find that I am not the only old-fashioned dentist that is wearing out; the other two older men here are on the same road, and they are good dentists. In the past six months I have had three old dentists from different parts call on me, and they said, 'We have sold out; we had to because we saw the wave coming.' And I asked how? And they said that they saw most of the dentistry was among the young people, and they would go to the young dentist even if he were not any better or did poorer dental work than we do. And I know here the people seem to think that the young man just ground out of the mill is the man. They are getting the business and that is the size of the case.

"Without doing any advertising except a card in the papers I took a P. G. course at ———— three years ago, and it did not do much good. If I could sell out my city property here I would take a new location, even if I am past the 50-

year mark. A man does not like to sit around his office and see the other fellow getting the business. So you can see where I am. What can be done? Please let me hear from you on the subject.

"Yours truly,

"F. H. S."

Let us take up this case and see what is the cause for this man's troubles. Let us ask him a few questions. What have you done in the thirty years you have been a resident of that city? Have you been an honorable, upright citizen? Have you played to the better element of your community? Have you kept your hands out of dirty politics? Have you howled for personal liberty, that a man may do as he pleases regardless of the welfare of the neighbors? Have you tried to help the rising generation to better things than booze, Sunday desecration, disobedience to law and order?

I ask these questions. I want to know what kind of a man he is in his city.

"I find that I am not the only old fashioned dentist." "Old fashioned dentist." Why are you the old-fashioned dentist? Is there anyone to blame but yourself? Are you one of those that proclaim, the no breakfast plan for me, and then, if I don't eat breakfast I can bum around as much as I please at night for I don't have to get up early for breakfast. I don't eat breakfast and I'll just stay abed as long as I please. I'll get to the office on time alright. Grant that he does as to the office hours, alright. but the assistant has been there. You lounge into your expensive office some where about the middle of the morning; you fiddle around the chair a few hours; go to lunch, and leave for the day early in the afternoon, and wonder why you don't succeed.

I mention these things that he who listens to my voice may sit up and take notice.

Why are you the old-fashioned dentist? Have you kept up with the times? Are you a member of your local dental society? Your district dental society? Your state dental society? Have you attended every meeting in each of these societies? Have you given any clinics or written any papers for your home or state society? Have you participated in the discussions of papers of either home or state societies? Have you ever asked another dentist whom you considered way up in the business when he arose to explain a certain piece of work, how a particular part was done, or were you afraid to expose your ignorance? Have you found anything good for you and for the other dentists as well, and taken it into the society meeting and told the whole push all about it? Or have you kept it to your own measly self?

I call your attention to these things that he who reads may ponder.

You are an old-fashioned dentist if you do not take a vacation. You will take some kind of a vacation either voluntary or involuntary. You old-fashioned dentist, you are taking your involuntary vacation now and the worst of it is you will never get back. You know what a vacation does for you? The best advertisement. People talk about you; how prosperous you are; how hard worked you are; just had to take a rest; he's a fine dentist but works too hard; glad to know he's taking a little time off.

I like my vacation all at one time rather than a run away vacation, a day here, two days at another time. When you are at home, be at home; when away, be away. I like the month of August best, and the whole month. Last year I took two months, this year six weeks. Dont' tell me you lose business by it. I do just as much business when I am away a month or two as when I used to stay home all the time, and I think more.

"And I took a P. G. course." "Hully Gee," what's that? Oh, I know. He went to the city and spent $50.00 for a course, and $50.00 for board and room, and $100.00 bumming around and left his wife at home making excuses for his not being at home when $25.00 would have taken him to the state society meeting and the $100.00 would have given the whole family a vacation and made everybody happy.

"If I could sell my city property I would move to another location." Why? Is your bad work coming back to torture you? Can't you make that upper plate stick without hurting? Can't you stop that jumping up and down of that lower plate? Are you still charging fifty cents for amalgam fillings, and gold, one dollar up? Are you the same dentist you were twenty-five years ago, without any improvement? If you are, no wonder the up to date dentist is getting the business. And you sold out because you saw the wave coming, and you let it come. A dentist fifty years of age and no business. Preposterous! Where is the trouble?

The questions I have asked will give you some idea of what I think about it, and I think the gentlemen who are selected to discuss this paper will add something more.

It is not necessary for me to say to you that I am a firm believer in the text, "As a man thinketh in his heart, so he is."

All of us carry around in our heads ideas of some kind, good, bad, or indifferent. I want to tell you what kind of thoughts to hold and what kind will prevent just such conditions as poor Dr. F. H. S. complains about.

Most people have three kinds of troubles. Those they have had; those they have now; and those they expect to have. The latter are the kind that give the most uneasiness. As for me, I dropped the past and my intimate friends know that I had many; and as for me now; look at me, do I look like a man filled with sorrow, a man carrying a heavy burden, a man down and out? Not on your life. And right here before I forget it. Don't let me hear any of you boys that are now about the thirty-year mark make such a howl twenty years hence. If you have no sand take some.

The one needed mental attitude is self assertion and self-reliance. When one relies upon himself he will express himself and will declare, "I can." Oh, the need of this faith, Emerson's "Self-Reliance" should be the textbook in every home. From the cradle up, one should be taught, "I can." This is the real text of life every young person should learn, and realize to trust thyself. Oh, ye sick, weak, uhappy and discouraged, listen to the call. Hold the thought of health, strength, happiness, and prosperity and let this be your motto, "I shall ask, I shall receive, I shall give."

### DISCUSSION BY DR. M. R. HARNED.

Dr. Cormany has given us a characteristic paper, just what we would expect from our smiling countenanced, good feeling, rotund, "Sunny Jim." Full of sense, faith and helpfulness. He need make no apology for presenting a paper on something other than a strictly technical subject, and when it comes to a practical subject, how could one be chosen that could be of more vital importance than how to avoid arriving at the pathetic situation wherein Dr. F. H. S. finds himself—that is if he F. H. S. really is—as bad off. and despondent as he thinks himself to be.

The sermon the author preaches to young dentists, taking this incident as a text, is timely and will be useful to each exactly in proportion as it is absorbed and acted upon by him. But how about he of the pathetic incident, wherein does he find the desired information, the magic word or touch which shall change him from a failure to a success? Unfortunately he can not go back thirty years, to the time he started to practice and thus profit by this advice and he is too old to get thoroughly into the civil, social or political life of his community if he has failed to do so previously.

To be sure, there is the advice of a Cormany, "to believe in himself, to assert himself, to ask, to receive, and to give." It is along the line of this thought though in more homely language, that the point may be made that Dr. F. H. S. probably has a serious attack of the blues, perhaps caused by an over indulgence in eating pork. If that be the case 'tis no wonder it has affected, not only his health but his pocketbook also.

I believe dentistry is a profession for the young man and that it is principally filled with young men, and probably it will always be so.

What percent of elderly men do we see at dental gatherings? From 1 to 10 percent possibly, and this proportion will about hold good throughout in active practice.

The active money making period in the dentist's career is from 25 to the 50th year in his life, and at the latter age he need not expect in the natural order of things, that his individual earnings are to increase year by year as his practice should have been doing many of the years previously. It is well that it be so, for he would have scarcely the time or energy to attend to it were it otherwise.

The majority of dentists at that age have and should have, other things which require some time and attention, and which either assist or are expected to assist, in producing an income. Orange groves, fruit ranches, farms or city property, gold mines, Mexican plantations, oil wells, or other kinds of present or future investments.

Many are more or less engaged in literary, educational, mercantile or manufacturing interests. At that time in life, he ought at least to have a home paid for, and if he has not been able to do that much he probably has been woefully deficient in ordinary business tactics.

Even F. H. S. has "city property" which he remarks may not be salable. Well, if it be not rentable either, why not raise potatoes upon it, if it be vacant lots? That would help some, both in occupation and income.

This appeal suggests considerably to me, that he is looking for assistance to come to him from the outside rather than from the inside, that is from himself. And yet, he would scarcely expect the editor of the BRIEF to go to his town and take the patients by main force from the crowded rooms of his young competitors and transfer them to his vacant chair.

He also complains that the young dentist just ground out gets all the business in his town. Strange what a difference there is in localities! I did not realize at the time —nor since—that I was doing all the business in the town the year after graduating.

To the majority of men who have been confined to the exacting nervous strain of the dental profession for 20, 30 or 40 years, there have probably come many thoughts of some avocation into which he would like to enter and spend the later years of his life; something broader, freer, further removed from the vexations of pleasing the public, and into which life may come more of God's pure air and sunshine, than is wont to enter into the ordinary dentists' apartments.

Therefore it is not strange that at 50 and 60 years of age. so many get out of active practice, neither is it strange that there is a strong tendency on the part of the public to patronize the younger and more energetic.

Speaking about annoying troubles, some one has truly said, there are two kinds about which we should not worry, those which we can help, and those which we can't help. For we should remedy those which we possibly can and with Dr. Cormany forget the others.

Really it would not matter upon what subject Dr. Cormany started to write or speak, toward the end of his paper or address he would get right into this "sand," "can" and "believe" business.

I dare say the paper he wrote upon prosthetic dentistry, ended the same way. Well, it is all right too, inspiring to any one, no matter of what profession, occupation or previous condition of servitude. He is a very healthy appearing example of the effects of his own medicine.—*Dental Review*—March 1911.

